# Author Correction: Recurrent *PTPRT/JAK2* mutations in lung adenocarcinoma among African Americans

**DOI:** 10.1038/s41467-020-14448-0

**Published:** 2020-01-30

**Authors:** Khadijah A. Mitchell, Noah Nichols, Wei Tang, Jennifer Walling, Holly Stevenson, Marbin Pineda, Roxana Stefanescu, Daniel C. Edelman, Andrew T. Girvin, Adriana Zingone, Sanju Sinha, Elise Bowman, Emily L. Rossi, Rony F. Arauz, Yuelin Jack Zhu, Justin Lack, Elizabeth Weingartner, Joshua J. Waterfall, Sharon R. Pine, John Simmons, Paul Meltzer, Bríd M. Ryan

**Affiliations:** 1 Laboratory of Human Carcinogenesis, Center for Cancer Research, National Cancer Institute, Bethesda, MD 20892 USA; 2 Genetics Branch, Center for Cancer Research, National Cancer Institute, Bethesda, MD 20892 USA; 3 Palantir Technologies, 1025 Thomas Jefferson St, Washington, DC, 20007 USA; 4 Cancer Data Science Laboratory, Center for Cancer Research, National Cancer Institute, Bethesda, MD 20892 USA; 5 NIAID Collaborative Bioinformatics Resource, National Institute of Allergy and Infectious Diseases, National Institutes of Health, Bethesda, MD 20892 USA; 6 Advanced Biomedical Computational Science, Frederick National Laboratory for Cancer Research sponsored by the National Cancer Institute, Frederick, MD 21702 USA; 7 Personal Genome Diagnostics, Baltimore, MD 21124 USA; 8 Rutgers Cancer Institute of New Jersey, Robert Wood Johnson Medical School, Rutgers, The State University of New Jersey, New Brunswick, NJ 08854 USA

**Keywords:** Cancer genomics, Lung cancer

Correction to: *Nature Communications* 10.1038/s41467-019-13732-y, published online 16 December 2019.

The original version of this Article contained an error in the spelling of the author Joshua J. Waterfall, which was incorrectly given as Josh Waterfall. This has now been corrected in both the PDF and HTML versions of the Article.

