# Supplementary material for: Recurrent PTPRT/JAK2 mutations in lung adenocarcinoma among African Americans
Source: Nat Commun. 2019 Dec 16;10:5735. doi: 10.1038/s41467-019-13732-y (PMC6915783; doi:10.1038/s41467-019-13732-y)
Supplement: Supplementary file 2 — Supplementary Information [file 41467_2019_13732_MOESM2_ESM.docx]

**Supplementary Information**

“**Recurrent *PTPRT/JAK2* Mutations in Lung Adenocarcinoma among African Americans**”

Mitchell *et al.*


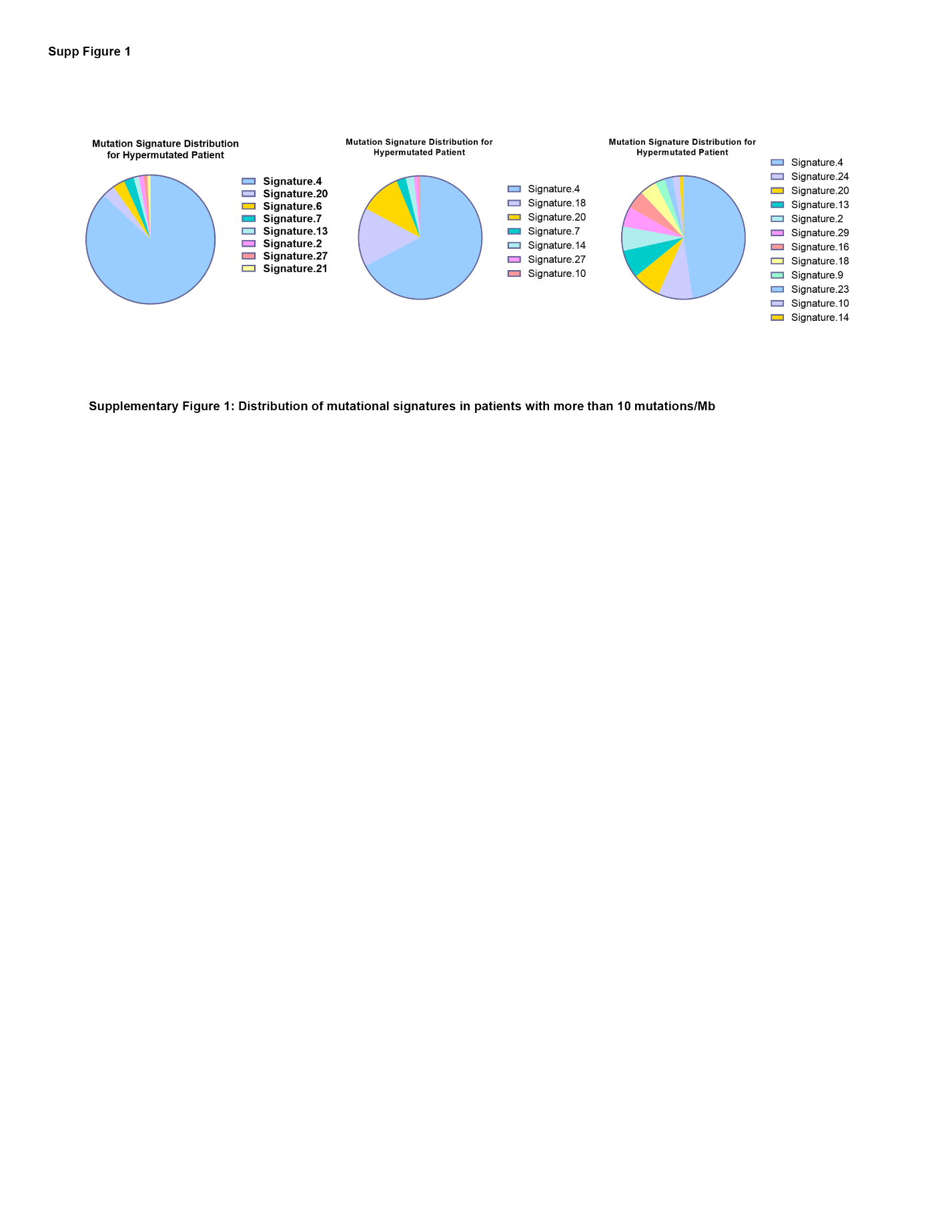


*
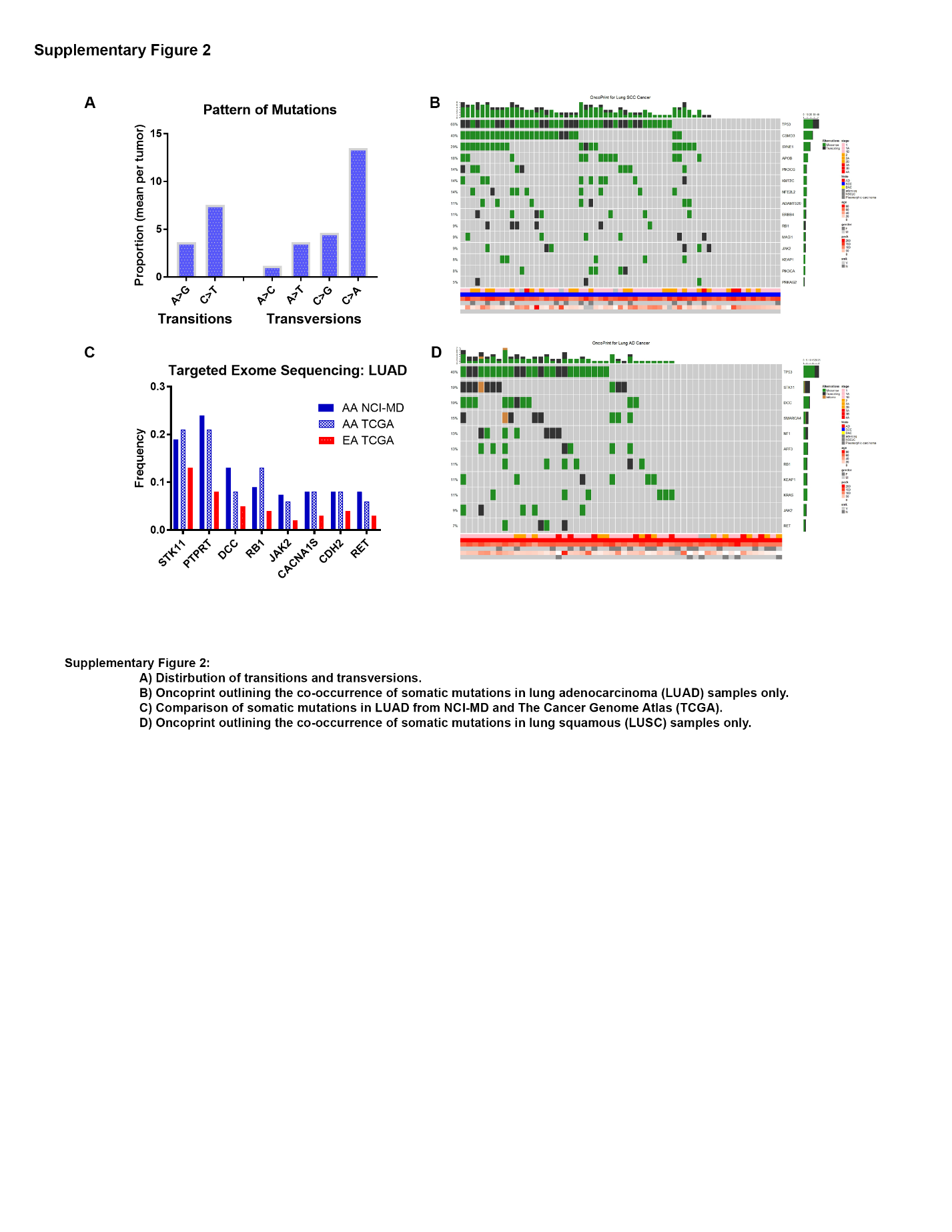
*

*
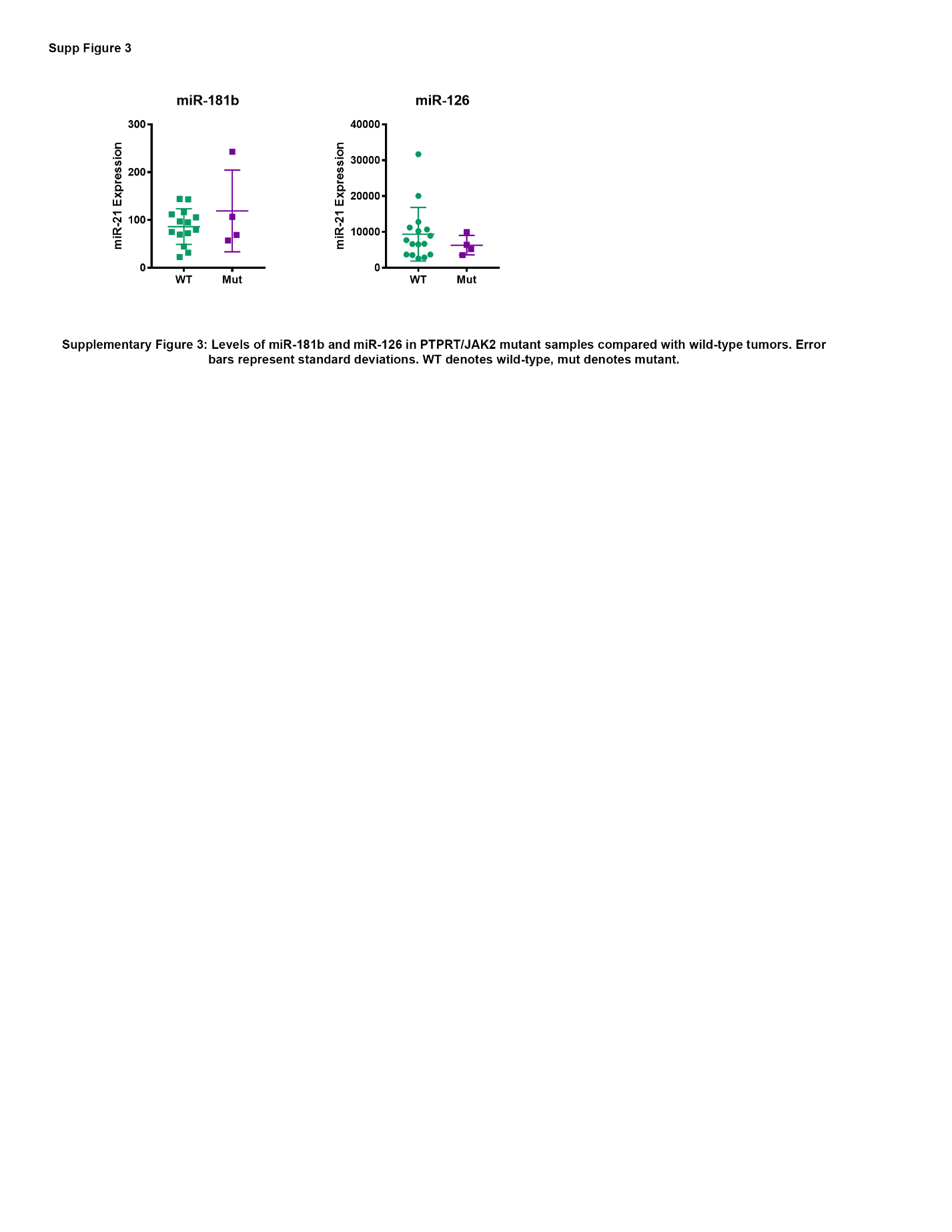
*

**Supplementary Table 1**: Characteristics of the study population

|  | | | |  |  |  |  |
| --- | --- | --- | --- | --- | --- | --- | --- |
| **Characteristic** | **NCI-MD** | **NCI-MD** | **NCI-MD** | **TCGA** | **TCGA** | **TCGA** | **TCGA** |
|  | **Targeted** | **WES** | **WES** | **LUAD** | **LUAD** | **LUSC** | **LUSC** |
|  | **(AA)** | **(AA)** | **(EA)** | **(AA)** | **(EA)** | **(AA)** | **(EA)** |
| **Race** |  |  |  |  |  |  |  |
| African American | 129 | 19 | 0 | 52 | 0 | 27 | 0 |
| European American | 0 | 0 | 31 | 0 | 381 | 0 | 331 |
| **Gender** |  |  |  |  |  |  |  |
| Male | 92 (71%) | 13 (68%) | 13 (42%) | 23 (44%) | 171 (45%) | 16 (59%) | 239 (72%) |
| Female | 37 (29%) | 6 (32%) | 18 (58%) | 29 (56%) | 210 (55%) | 11 (41%) | 92 (28%) |
| **Smoking Status** |  |  |  |  |  |  |  |
| Never | 6 (5%) | 0 (0%) | 0 (0%) | 3 (6%) | 57 (15%) | 1 (4%) | 9 (3%) |
| Former | 38 (29%) | 7 (37%) | 12 (39%) | 30 (57%) | 279 (60%) | 15 (56%) | 210 (63%) |
| Current | 82 (64%) | 12 (63%) | 19 (61%) | 17 (33%) | 83 (22%) | 10 (36%) | 102 (31%) |
| Unknown | 3 (2%) | 0 | 0 | 2 (4%) | 12 (3%) | 1 (4%) | 10 (3%) |
| **Pack-years** (median, IQR) | 38 (25-60) | 40 (30-93) | 41 (22-66) | 30 (15-40) | 40 (25-54) | 50 (30-80) | 45 (30-60) |
| **Age** (mean ± SD) | 63 ± 8.5 | 64 (57-79) | 64 (61-69) | 60 ± 10.3 | 66 ± 9.8 | 67 ± 7.8 | 66 ± 8.9 |
| **Histology** |  |  |  |  |  |  |  |
| Adenocarcinoma | 54 (42%) | 6 (21%) | 22 (71%) | 51 | 359 | 0 | 0 |
| Squamous Cell Carcinoma | 65 (50%) | 9 (47%) | 7 (23%) | 0 | 0 | 27 | 331 |
| BAC | 7 (5%) | 0 (0%) | 0 (0%) | 1 | 22 | 0 | 0 |
| Other | 3 (2%) | 4 (47%) | 2 (6%) | 0 | 0 | 0 | 0 |
| **Stage** |  |  |  |  |  |  |  |
| I | 73 (57%) | 9 (47%) | 13 (42%) | 26 (50%) | 212 (56%) | 15 (55%) | 166 (50%) |
| II | 35 (27%) | 2 (11%) | 8 (26%) | 15 (29%) | 88 (23%) | 5 (17%) | 112 (37%) |
| III | 13 (10%) | 4 (21%) | 5 (16%) | 7 (13%) | 60 (16%) | 7 (26%) | 46 (10%) |
| IV | 1 (1%) | 1 (5%) | 0 (0%) | 2 (4%) | 16 (4%) | 0 | 3 (1%) |
| Unknown | 7 (5%) | 3 (16%) | 5 (16%) | 2 (4%) | 5 (1%) | 0 | 4 (1%) |
|  |  |  |  |  |  |  |  |
| SD denotes standard deviation, IQR denotes interquartile range, BAC denotes bronchioalveolar carcinoma | | | | | | |  |
