# Supplementary material for: Recurrent PTPRT/JAK2 mutations in lung adenocarcinoma among African Americans
Source: Nat Commun. 2019 Dec 16;10:5735. doi: 10.1038/s41467-019-13732-y (PMC6915783; doi:10.1038/s41467-019-13732-y)
Supplement: Supplementary file 3 — Description of Additional Supplementary Files [file 41467_2019_13732_MOESM3_ESM.pdf]

Description of Additional Supplementary Files

File Name: Supplementary Data 1
Description: Annotation of Gene Expression Signatures.

File Name: Supplementary Data 2
Description: List of genes included in targeted sequencing platform.

File Name: Supplementary Data 3
Description: List of genes with no somatic mutations among 129 tumor/normal pairs.

File Name: Supplementary Data 4
Description: Summary of somatic mutation data from targeted exome sequencing.

File Name: Supplementary Data 5
Description: Summary of somatic mutation data from targeted exome sequencing (post filtering for likely protein modifying variants).

File Name: Supplementary Data 6
Description: Summary of mutational signature analysis.

File Name: Supplementary Data 7
Description: Frequency of known driver gene mutations in LUAD and LUSC in African Americans and European Americans.

File Name: Supplementary Data 8
Description: Analysis of mutual exclusivity for PTPRT, JAK2 and key oncogenes/tumor suppressors in LUAD.

File Name: Supplementary Data 9
Description: Summary of somatic mutations following filtration for potentially functional variants from whole exome sequencing in the NCI-MD study.

File Name: Supplementary Data 10
Description: Predicted PTPRT and JAK2 fusion genes from WES data in NCI-MD Case Control study.

File Name: Supplementary Data 11
Description: Differential drug susceptibility based on PTPRT (top) and JAK2 (bottom) mutation status using the Cancer Dependency Map.

File Name: Supplementary Data 12
Description: Sequencing statistics for the targeted sequencing panel.
